# Kawasaki disease and 13-valent pneumococcal conjugate vaccination among young children: A self-controlled risk interval and cohort study with null results

**DOI:** 10.1371/journal.pmed.1002844

**Published:** 2019-07-02

**Authors:** Meghan A. Baker, Bethany Baer, Martin Kulldorff, Lauren Zichittella, Rebecca Reindel, Sandra DeLuccia, Hana Lipowicz, Katherine Freitas, Robert Jin, W. Katherine Yih

**Affiliations:** 1 Department of Population Medicine, Harvard Pilgrim Health Care Institute and Harvard Medical School, Boston, Massachusetts, United States of America; 2 Department of Medicine, Brigham and Women’s Hospital, Boston, Massachusetts, United States of America; 3 Center for Biologics Evaluation and Research, Food and Drug Administration, Silver Spring, Maryland, United States of America; 4 Division of Pharmacoepidemiology and Pharmacoeconomics, Brigham and Women’s Hospital, Boston, Massachusetts, United States of America; Epicentre, FRANCE

## Abstract

**Background:**

Kawasaki disease is an acute vasculitis that primarily affects children younger than 5 years of age. Its etiology is unknown. The United States Vaccine Safety Datalink conducted postlicensure safety surveillance for 13-valent pneumococcal conjugate vaccine (PCV13), comparing the risk of Kawasaki disease within 28 days of PCV13 vaccination with the historical risk after 7-valent PCV (PCV7) vaccination and using chart-validation. A relative risk (RR) of 2.38 (95% CI 0.92–6.38) was found. Concurrently, the Food and Drug Administration (FDA) conducted a postlicensure safety review that identified cases of Kawasaki disease through adverse event reporting. The FDA decided to initiate a larger study of Kawasaki disease risk following PCV13 vaccination in the claims-based Sentinel/Postlicensure Rapid Immunization Safety Monitoring (PRISM) surveillance system. The objective of this study was to determine the existence and magnitude of any increased risk of Kawasaki disease in the 28 days following PCV13 vaccination.

**Methods and findings:**

The study population included mostly commercially insured children from birth to <24 months of age in 2010 to 2015 from across the US. Using claims data of participating Sentinel/PRISM data-providing organizations, PCV13 vaccinations were identified by means of current procedural terminology (CPT), Healthcare Common Procedure Coding System (HCPCS), and National Drug Code (NDC) codes. Potential cases of Kawasaki disease were identified by first-in-365-days International Classification of Diseases 9th revision (ICD-9) code 446.1 or International Classification of Diseases 10th revision (ICD-10) code M30.3 in the inpatient setting. Medical records were sought for potential cases and adjudicated by board-certified pediatricians. The primary analysis used chart-confirmed cases with adjudicated symptom onset in a self-controlled risk interval (SCRI) design, which controls for time-invariant potential confounders. The prespecified risk interval was Days 1–28 after vaccination; a 28-day-long control interval followed this risk interval. A secondary analytic approach used a cohort design, with alternative potential risk intervals of Days 1–28 and Days 1–42. The varying background risk of Kawasaki disease by age was adjusted for in both designs. In the primary analysis, there were 43 confirmed cases of Kawasaki disease in the risk interval and 44 in the control interval. The age-adjusted risk estimate was 1.07 (95% CI 0.70–1.63; *p* = 0.76). In the secondary, cohort analyses, which included roughly 700 potential cases and more than 3 million person-years, the risk estimates of potential Kawasaki disease in the risk interval versus in unexposed person-time were 0.84 (95% CI 0.65–1.08; *p* = 0.18) for the Days 1–28 risk interval and 0.97 (95% CI 0.79–1.19; *p* = 0.80) for the Days 1–42 risk interval. The main limitation of the study was that we lacked the resources to conduct medical record review for all the potential cases of Kawasaki disease. As a result, potential cases rather than chart-confirmed cases were used in the cohort analyses.

**Conclusions:**

With more than 6 million doses of PCV13 administered, no evidence was found of an association between PCV13 vaccination and Kawasaki disease onset in the 4 weeks after vaccination nor of an elevated risk extending or concentrated somewhat beyond 4 weeks. These null results were consistent across alternative designs, age-adjustment methods, control intervals, and categories of Kawasaki disease case included.

## Abstract 2

W. Katherine Yih and colleagues reveal no association between 13-valent pneumococcal conjugate vaccination in children and Kawasaki disease onset

## Author summary

**Why was this study done?:**

- Kawasaki disease is an acute vasculitis that primarily affects children younger than 5 years of age. It is the leading cause of acquired heart disease in children in developed countries, and its etiology is unknown.
- Vaccine Safety Datalink investigators found a nonstatistically significant elevated risk of Kawasaki disease after immunization with 13-valent pneumococcal conjugate vaccine (PCV13).
- Spontaneous reports of Kawasaki disease after PCV13 have been received by the Vaccine Adverse Event Reporting System.
- On the basis of these observations, the Food and Drug Administration (FDA) requested that Kawasaki disease after PCV13 vaccination in young children be studied in the claims-based Sentinel/Postlicensure Rapid Immunization Safety Monitoring (PRISM) system.

**What did the researchers do and find?:**

- We used health insurance claims data of children 0 to <2 years of age from 6 Sentinel/PRISM Data Partners from 2010 to 2015.
- Board-certified clinicians used medical records to adjudicate the Kawasaki disease cases for the primary study design. The adjudicators were blinded to the cases’ vaccination status.
- The primary analysis used confirmed cases in a self-controlled design with a risk interval of Days 1–28 after vaccination, adjusting for the varying risk of Kawasaki disease by age.
- A number of secondary analyses were conducted, including cohort analyses, analyses including cases of different levels of diagnostic certainty, analyses with alternative risk and comparison intervals, analyses using different populations for age adjustment, and a temporal scan statistical analysis.
- More than 6 million doses of PCV13 were administered to the study population.
- Eighty-seven cases of confirmed Kawasaki disease were included in the primary analysis, which did not find evidence of an increased risk of Kawasaki disease in vaccinees (relative risk [RR]: 1.07; 95% CI 0.70–1.63; *p* = 0.76).
- Results of all secondary analyses were also null.

**What do these findings mean?:**

- No evidence was found of an elevated risk of Kawasaki disease in the 28 days after PCV13 vaccination nor of an elevated risk extending or concentrated somewhat beyond 28 days.

## Introduction

In 2000, the Food and Drug Administration (FDA) licensed the first pneumococcal conjugate vaccine (PCV), 7-valent PCV (PCV7; Prevnar; Wyeth), to protect young children against invasive disease caused by any of 7 serotypes of *Streptococcus pneumoniae*. Inclusion of PCV7 in the recommended child immunization program at 2, 4, 6, and 12–15 months of age resulted in decreased rates of invasive pneumococcal disease [1,2]. In early 2010, the FDA licensed a second vaccine, PCV13 (Prevnar 13; Wyeth), with the same vaccination schedule as PCV7, to replace PCV7 and protect against 6 additional serotypes that cause invasive pneumococcal disease in young children [3].

Although prelicensure trials of PCV7 and PCV13 found no increased risk of serious adverse events [4], postlicensure surveillance raised questions about a possible association between PCV13 and Kawasaki disease, an acute, self-limited vasculitis with a predilection for the coronary arteries and the leading cause of acquired heart disease in children in the United States. When Centers for Disease Control and Prevention (CDC)-sponsored Vaccine Safety Datalink investigators monitored the safety of the PCV13 vaccine during the first 2 years of life with respect to 8 health outcomes, a statistically significant result for Kawasaki disease after PCV13 was identified in the second of 12 group-sequential tests. The investigators conducted an end-of-surveillance analysis restricted to chart-confirmed cases and found a nonstatistically significant relative risk (RR) of 2.38 (95% CI 0.92–6.38) in the 0 to 28 days following vaccination with PCV13 compared with after PCV7 [5]. The FDA completed a safety review of the first 18 months of licensure of PCV13 under the FDA Amendment Act of 2007 Section 915, which included an analysis of the Vaccine Safety Datalink study results as well as an evaluation of the Vaccine Adverse Event Reporting System proportional reporting ratios for Kawasaki disease. The review resulted in an FDA Postmarket Safety Evaluation Summary posting that stated that there had been reports of Kawasaki disease following administration of PCV13 and that the FDA intended to initiate a larger study [6]. This report describes the now-completed study conducted in the Postlicensure Rapid Immunization Safety Monitoring (PRISM) program, a component of the FDA-sponsored Sentinel Initiative [7].

## Methods

### Study population and data sources

Six Sentinel/PRISM Data Partners, all in the US, contributed data: Aetna; Harvard Pilgrim Health Care; HealthCore (Anthem); Humana; OptumInsight LifeSciences; and Vanderbilt University School of Medicine, Department of Health Policy. Further details about the Data Partners are available on the Sentinel website [8]; most of the population represented by these Data Partners is commercially insured and not concentrated in any particular region of the country, although a small portion is covered by Medicaid in Tennessee. The study population included children less than 2 years of age who were members of any of the 6 Data Partners during 2010 to 2015 and who met 1 of 2 other enrollment criteria: (1) were exposed to at least one dose of any PCV vaccine and continuously enrolled at the Data Partner from birth through at least 84 days after their first dose of any PCV vaccine, or (2) were unexposed to any PCV vaccine and were continuously enrolled at the Data Partner from birth through at least 144 days of age (60 days, the time that many infants would receive dose 1, plus 84 days), with at least one documented healthcare visit between 14 and 150 days of age (i.e., up to 5 months of age). Gaps of up to 45 days between birth and the start of enrollment were allowed.

To model Kawasaki disease risk by age for the age adjustment implemented in the primary analysis, we used the Kids’ Inpatient Database for 2009 of the Healthcare Cost and Utilization Project (HCUP), Agency for Healthcare Research and Quality [9].

### Exposure identification

PCV13 was identified by the Current Procedural Terminology (CPT) code 90670 and the National Drug Codes 00005197101, 00005197102, 00005197104, and 00005197105 since January 1, 2010. Unspecified PCV was identified by the CPT code 90669 and Healthcare Common Procedure Coding System codes G0009 and S0195. Unspecified PCV vaccine since September 1, 2010, was assumed to be PCV13, based on the date of approval for PCV13 (February 24, 2010) and the fact that by July 2010 Pfizer reported that >90% of its private shipments of PCVs were for PCV13 [10]. Previous work in the Sentinel/PRISM system has found that most routinely administered vaccines are well captured by claims data [11].

### Outcome identification

Potential cases of Kawasaki disease were identified by the International Classification of Diseases 9th revision (ICD-9) code 446.1 and the International Classification of Diseases 10th revision (ICD-10) code M30.3 (acute febrile mucocutaneous lymph node syndrome) in the inpatient setting in any position (e.g., primary diagnosis, secondary diagnosis, etc.). (More than 95% of patients with an initial diagnosis of Kawasaki disease are hospitalized [12].) Only the first code in 365 days in the inpatient setting for patients at least 365 days of age or the first ever code in the inpatient setting for those under 365 days of age was considered, in order to exclude follow-up visits for Kawasaki disease.

### Medical record review

Medical records of all potential Kawasaki disease cases (as ascertained by the algorithm) occurring during the 70 days after PCV13 vaccination were requested. In addition, records of potential cases without any known PCV vaccination were sought. We requested the inpatient hospitalization record associated with the healthcare claim with Kawasaki disease diagnosis code, as well as an outpatient follow-up visit for Kawasaki disease, including records of echocardiograms and angiograms.

Three board-certified pediatricians served as case adjudicators. Twenty cases were double-adjudicated, using prespecified classification rules. Once all discrepancies were resolved and classification rules refined, the chart review process continued with a single review of all remaining cases. The adjudicators were blinded to potential cases’ vaccination history.

Diagnosis of Kawasaki disease was based on the American Heart Association diagnosis guidelines and the CDC case definition [13,14]. Cases of confirmed (“Level 1”) Kawasaki disease were defined as those meeting one of the following criteria: (1) ≥4 principal features and a fever (≥38.0°C) persisting ≥5 days or until administration of intravenous immunoglobulin if given before the fifth day of fever, or (2) <4 principal features, fever (≥38.0°C) of any duration, and coronary artery disease (aneurysm or dilation) detected by either echocardiography or coronary angiography. The 5 principal clinical features are (1) changes in the extremities (erythema of palms or soles, edema of hands or feet, and/or periungual desquamation in the subacute phase), (2) polymorphous exanthem rash, (3) bilateral conjunctival injection without exudates, (4) changes in lips and oral cavity (inflamed lips or throat, strawberry tongue, or dry/cracking lips), and (5) cervical lymphadenopathy (at least one lymph node ≥ 1.5 cm in diameter) [13,15].

Cases of “possible” (“Level 2”) Kawasaki disease were defined as those having evidence of 2 or 3 principal features and ≥5 days of fever. The “possible” Kawasaki disease category was of interest, because some principal clinical features are frequently absent in young infants [13], and a large proportion of our study population was under 12 months of age. “Inconclusive” Kawasaki disease was defined as 1 principal feature and ≥5 days of fever.

During their record review, adjudicators determined the timing of symptom onset relative to the date of the coded diagnosis.

For cases of Kawasaki disease without a prior PCV vaccination history in the administrative claims data, the immunization record was sought from the child’s primary care provider for verification.

### Risk intervals

The primary risk interval (or risk “window”) used in the analyses was Days 1–28 after any dose of PCV13, where Day 0 was the day of vaccination. This risk interval was also used in the Vaccine Safety Datalink sequential analysis of PCV13 [5] and is indirectly supported by evidence that, among siblings, more than one-half of second Kawasaki disease cases in each family developed within 10 days of onset of symptoms in the first case, a finding consistent with a shared environmental trigger (or consecutive triggering infections with short incubation periods) and a relatively short latency period of days or weeks rather than months after exposure [16]. In a secondary analysis, we considered a postvaccination risk interval of Days 1–42. The 42-day interval allowed us to address any concerns that, if PCV13 were associated with an increased risk of Kawasaki disease, the true period of increased risk might go beyond the first 28 days.

### Statistical analyses

#### Overview

In the statistical analyses, we sought to examine and control for the confounding effect of age, because both PCV13 vaccination and the risk of Kawasaki disease are agedependent, the risk varying by small increments of age [17]. The analyses consisted of both age-adjusted and age-unadjusted self-controlled risk interval (SCRI) analyses using logistic regression, as well as age-adjusted cohort analyses using unconditional Poisson regression. One of the cohort analyses used a risk interval of Days 1–28, and the other used a risk interval of Days 1–42. No analyses specific to dose number were conducted, i.e., all doses were pooled in analysis. These analyses are summarized in Table 1 and are discussed in greater detail below.

**Table 1:** Statistical analysis methods used.

1° versus 2°	Design	Regression	Age adjustment	Risk window
Primary	SCRI	Logistic	Offset term (from HCUP data)	Days 1–28
Secondary	SCRI	Logistic	None	Days 1–28
Secondary	Cohort	Unconditional Poisson	Internal, from study population	Days 1–28
Secondary	Cohort	Unconditional Poisson	Internal, from study population	Days 1–42

**Abbreviations:** HCUP, Healthcare Cost and Utilization Project; SCRI, self-controlled risk interval

#### SCRI analyses

A major advantage of the SCRI design is that it inherently controls for all fixed (non–time-varying) potential confounders such as sex, race/ethnicity, and chronic predisposing conditions, by virtue of each subject serving as his/her own control. The null hypothesis with this design is that the risk of Kawasaki disease is the same on an average day in the risk interval as on an average day in the control interval. Adjustment for time-varying confounders such as age must be made explicitly. We used a risk interval of Days 1–28 post-vaccination and a 28-day-long control interval following this risk interval. The prespecified control intervals were Days 29–56 for Doses 1 and 2 and Days 43–70 for Doses 3 and 4. We considered the latter interval to be preferable due to uncertainty about the true period of vaccine-associated risk and the possibility that it might extend beyond 28 days after vaccination. However, the recommended ages of 2, 4, and 6 months for receipt of Doses 1, 2, and 3, respectively, made it necessary to fit the control interval for Doses 1 and 2 into a 2-month period so as to avoid the control interval of one dose overlapping the risk interval of a subsequent dose. Confirmed and possible cases were assigned to risk or control intervals according to the timing of their adjudicated symptom onset.

Because the vaccinated potential cases selected for chart review included only those ascertained out to 70 days post-vaccination in the administrative data, it was possible that some true cases with hospital admission dates beyond Day 70 but symptom onset within 70 days were missed. To address this, we conducted an additional set of analyses using Days 29–56 as the control interval for all 4 doses. We limited the cases analyzed in these post hoc analyses to those for whom the Kawasaki disease hospital admission date minus the symptom onset date was ≤14 days, the difference between Day 70 and Day 56. The reason for this restriction was to avoid bias—without the restriction, all other things being equal, there would have tended to be more confirmed cases in the risk interval than in the control interval, due to the earlier timing of the risk interval.

For the age-adjusted SCRI analyses, we first modeled the background risk of Kawasaki disease by age. To do this, we used Kawasaki disease counts from HCUP’s Kids’ Inpatient Database for 2009, which was the most recent year for which age by month was available. (A similar approach was used in the Sentinel/PRISM study of the newer-generation rotavirus vaccines and intussusception [18].) Using data for the age range of 2 to 35 months, we modeled Kawasaki disease occurrence by age, trying first-, second-, third-, fourth-, and fifth-order polynomial functions in successive Poisson models to determine the best-fitting function. The fourth-order function fit well according to the Akaike information criterion and according to visual comparison with the data, thus we did not resort to splines. Predicted values from this quartic polynomial function were used to calculate offset terms for the cases in the logistic regression analysis in order to adjust for the differential risk of Kawasaki disease according to age in the risk and control intervals. These estimates were based on 1,224 potential cases of Kawasaki disease identified from claims in the HCUP data set and were treated as known without error. Treating the background risk estimates as known without error has been shown to produce very similar results as when accounting for uncertainty in the estimates, as long as the number of cases in the population used to estimate the background risk is greater than the number of cases in the SCRI analysis, which is the case in this study. Although that work is unpublished, the general approach and findings are analogous to those in a published study by the same investigator [19].

#### Cohort analyses

**Fig 1:**
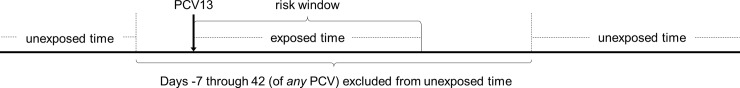
Illustration of segments of exposed and unexposed person-time for a PCV13-vaccinated child contributing person-time to the cohort analysis with the Days 1–28 risk interval. Person-time within the upward-pointing bracket was categorized as exposed, whereas person-time occurring outside of the downward-pointing bracket was categorized as unexposed. Person-time occurring during Days −7 through 0 and Days 29–42 of any PCV vaccination was not included in the analysis. Likewise, Kawasaki disease cases in the cohort analysis were categorized as exposed, unexposed, or excluded depending on the time segment in which they occurred. PCV13, 13-valent pneumococcal conjugate vaccine.

We used a cohort design with unconditional Poisson regression as a secondary analysis method, including cases and person-time from 2010 through a maximum of September 2015. Exposed person-time was defined as Days 1–28 or Days 1–42 after PCV13 vaccination, depending on the specific analysis (see Table 1, last 2 rows). Exposed cases were those occurring in the respective time period (Days 1–28 or 1–42). Unexposed person-time was defined as the time outside of the 7 days before, through the 42 days after, vaccination with either PCV13 or PCV7, including time from children not vaccinated with any PCV vaccine but with at least 1 documented healthcare visit. (We excluded the 7 days prior to vaccination in order to control for the healthy vaccinee effect and similar effects that could result from dependency of vaccination on one’s health condition in the week prior.) Unexposed cases were those occurring in any unexposed time. Fig 1 illustrates the categorization of exposed and unexposed person-time.

Data Partner, calendar year, sex, and age in weeks were included in the modeling. Age in weeks was modeled as a continuous variable; as with the HCUP data, we tried increasing orders of polynomial function (linear, quadratic, cubic, etc.) in successive models to determine the most appropriate, based on log-likelihood ratio, *p*-value, Akaike information criterion, and biologic plausibility. The quartic (fourth-order) function was selected for the final model.

Due to the restrictions applied to potential cases for chart review and the limited number of chart-confirmed cases available for modeling Kawasaki disease by age, we conducted the cohort analyses using potential cases based on the administrative data rather than restricting the analyses to the chart-confirmed cases. There was no systematic difference in chart-confirmation ratio by age. However, given the overall case-confirmation ratio of 68% (reported below), this use of all potential cases meant that there was some misclassification of the outcome. On conducting a chi-squared test, we found no statistically significant difference in case-confirmation ratio by exposure status, suggesting that the misclassification was nondifferential. The implications of this are discussed below.

#### Temporal scan analysis

To find any clustering of Kawasaki disease onsets within the 56 days after PCV13 vaccination, we used the temporal scan statistic, a self-controlled method that controls for multiple testing [20,21]. To avoid possible bias, we included only the 91 confirmed cases with onsets during Days 1–56 post-vaccination for whom the difference between the hospital admission date and adjudicated symptom onset date was ≤14 days. All potential risk windows during the 56-day follow-up period that were between 1 and 28 days in length, inclusive, were evaluated.

### Public health surveillance status and supporting information

The Sentinel Initiative is a public health surveillance activity [22], thus this study was not under the purview of institutional review boards.

The study protocol (S1 Protocol), STROBE checklist (S1 STROBE Checklist), and SCRI analysis datasets (S1 Data, S2 Data, S3 Data, and S4 Data) are provided as supporting information.

## Results

### Vaccine doses administered

A total of 6,177,795 doses of PCV13 vaccine were administered to the study population.

### Kawasaki disease cases

There were 206 potential cases of Kawasaki disease, all ascertained by the presence of the ICD-9 code 446.1, meeting the criteria for chart review. Medical records were obtained for 184 (89%) of these. Of the 184 cases for whom charts were obtained, 125 (68%) were determined by clinical adjudication to be confirmed Kawasaki disease, 29 (16%) were determined to be possible Kawasaki disease, 4 (2%) were considered inconclusive, 18 (10%) lacked the necessary information for adjudicators to make a determination, and 8 (4%) were ruled out. The case-confirmation proportion was thus 68% for Level 1 Kawasaki disease and 84% for Level 1 plus Level 2.

### SCRI analyses

In the SCRI logistic regression analyses that used the prespecified control windows, there were 43 confirmed cases in the risk window and 44 in the control window. No evidence of an elevation in risk was observed in either the (primary) HCUP–age-adjusted analysis or the unadjusted analysis—the RRs were 1.07 (95% CI 0.70–1.63; *p* = 0.76) and 0.98 (95% CI 0.64–1.49; *p* = 0.91), respectively. Adding in the possible Kawasaki disease cases did not qualitatively change these findings—there were 53 confirmed or possible cases in the risk window and 53 in the control window, for an RR of 1.09 (95% CI 0.75–1.60; *p* = 0.64) in the adjusted analysis and a RR of 1.00 (95% CI 0.68–1.46; *p* = 1.00) in the unadjusted analysis (Table 2).

**Table 2:** Results of SCRI analyses.

Age adjustment	Cases in RW	Cases in CW	KD level of diagnostic certainty*	RR (95% CI)	*p*-value
*With Doses 1 and 2 CW = Days 29–56 and Doses 3 and 4 CW = Days 43–70*					
HCUP data	43	44	Level 1	1.07 (0.70–1.63)	0.76
None	43	44	Level 1	0.98 (0.64–1.49)	0.91
HCUP data	53	53	Level 1 + 2	1.09 (0.75–1.60)	0.64
None	53	53	Level 1 + 2	1.00 (0.68–1.46)	1.00
*With all CWs = Days 29–56 and no cases for which (KD admit–KD onset) > 14 days*:					
HCUP data	41	50	Level 1	0.89 (0.59–1.34)	0.57
None	41	50	Level 1	0.82 (0.54–1.24)	0.35
HCUP data	50	61	Level 1 + 2	0.89 (0.61–1.29)	0.53
None	50	61	Level 1 + 2	0.82 (0.56–1.19)	0.30

*Level 1 = confirmed; Level 2 = possible

**Abbreviations:** CW, control window; HCUP, Healthcare Cost and Utilization Project; KD, Kawasaki disease; RR, relative risk; RW, risk window; SCRI, self-controlled risk interval

The post hoc SCRI logistic regression analyses that used Days 29–56 following vaccination as the control window for all doses and restricted cases for analysis to those in which the difference between the hospital admission date and adjudicated symptom onset date was ≤14 days produced similarly null results. There were 41 cases in the risk window and 50 in the control window, and the adjusted and unadjusted risk estimates for the confirmed cases were 0.89 (95% CI 0.59–1.34; *p* = 0.57) and 0.82 (95% CI 0.54–1.24; *p* = 0.35), respectively. When the possible cases were included with the confirmed cases, there were 50 cases in the risk window and 61 in the control window, giving adjusted and unadjusted risk estimates of 0.89 (95% CI 0.61–1.29; *p* = 0.53) and 0.82 (95% CI 0.56–1.19; *p* = 0.30), respectively (Table 2).

### Cohort analyses

The cohort for the analysis using the Days 1–28 risk window contained 80 potential Kawasaki disease cases (based on claims) in the risk window and approximately 474,000 exposed person-years. The cohort for the analysis using the Days 1–42 risk window contained 145 potential cases in that risk window and approximately 711,000 exposed person-years. Both data sets had 598 potential cases in unexposed time and 2.7 million person-years of unexposed person-time.

The risk estimates of potential Kawasaki disease in the risk window versus in unexposed time were 0.84 (95% CI 0.65–1.08; *p* = 0.18) for the Days 1–28 risk window and 0.97 (95% CI 0.79–1.19; *p* = 0.80) for the Days 1–42 risk window.

The nondifferential misclassification of the outcome entailed in not restricting the cohort analyses to chart-confirmed cases introduced noise, biasing the risk estimates toward the null. Given that the risk estimates were <1, unbiased estimates would have been somewhat lower than those observed.

### Temporal scan analysis

**Fig 2:**
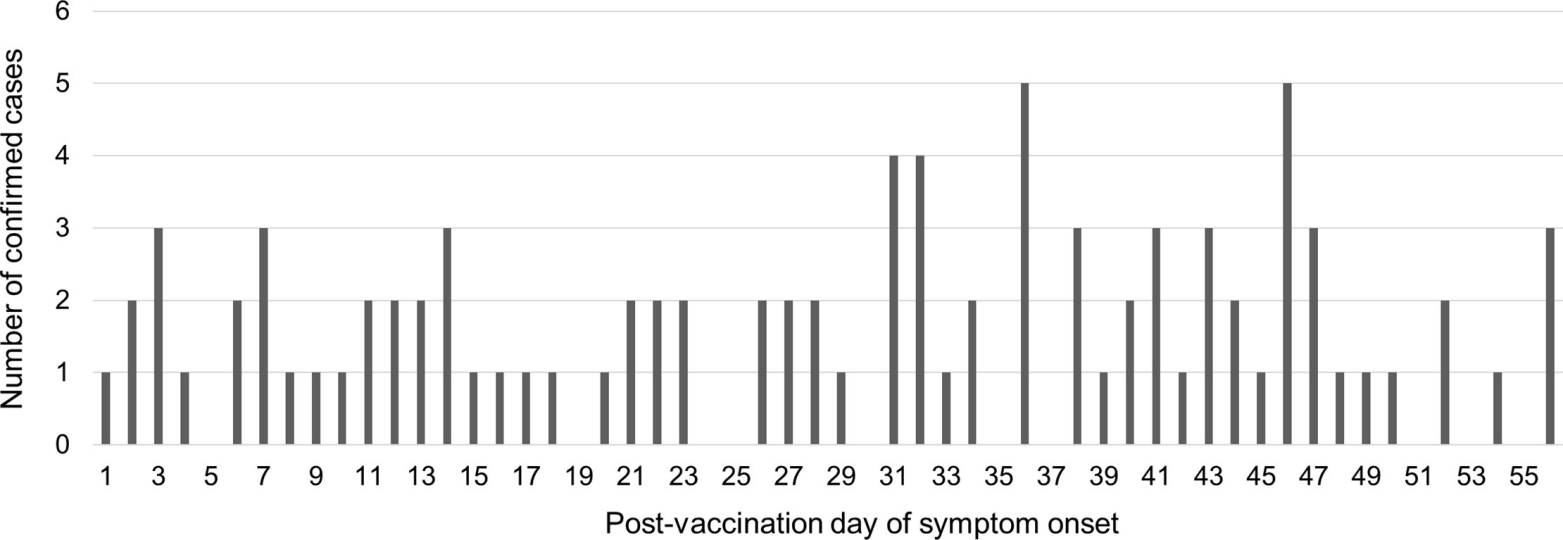
Temporal distribution of adjudicated Kawasaki disease symptom onsets for the 91 confirmed cases during Days 1–56 post–PCV13 vaccination for which the difference between the hospital admission date and adjudicated symptom onset date was ≤14 days. PVC13, 13-valent pneumococcal conjugate vaccine.

Fig 2 shows the temporal distribution of Kawasaki disease symptom onsets for the 91 confirmed cases during Days 1–56 post–PCV13 vaccination for which the difference between the hospital admission date and adjudicated symptom onset date was ≤ 14 days. The temporal scan statistical test found no statistically significant clustering of cases. The lowest *p*-value of any grouping was 0.34.

## Discussion

In this large study investigating the relationship between PCV13 vaccination and Kawasaki disease during the 1–28 days after vaccination, we found no evidence of an association. The study included 87 confirmed cases in the primary SCRI analysis and approximately 700 potential cases and more than 3 million person-years in the secondary cohort analyses. The results were consistently null across alternative methods of analysis and age adjustment, alternative control intervals, and alternative levels of diagnostic certainty included.

Our null results contrast with the elevated (albeit not statistically significant) point estimate of Kawasaki disease risk during Days 0–28 after PCV13 found by Vaccine Safety Datalink investigators (RR = 2.38; 95% CI 0.92–6.38), who used historical rates of Kawasaki disease after PCV7 for comparison [5]. We consider our results to be quite robust because of the size of the study, with 6 million doses; the self-controlled nature of the primary analysis; and the qualitatively similar results obtained in all our analyses, both primary and secondary.

The possibility that any true period of increased risk might extend or be concentrated somewhat beyond 28 days post-vaccination was taken into consideration in several ways: (a) the primary SCRI analysis, which used a control interval of Days 43–70 for Doses 3 and 4, (b) a cohort analysis using a risk interval of Days 1–42, and (c) a temporal scan statistical analysis to detect clustering of onsets in any 1- to 28-day-long period during Days 1–56. No evidence for an increased risk after vaccination was found in any of these analyses.

The main limitation of the study was that we lacked the resources to conduct medical record review for all 685 potential cases of Kawasaki disease during 2010–2015, and in applying criteria to limit the cases for chart review, we unintentionally excluded potential cases with hospital admission during Days 71–84 after vaccination. As a result, some true cases with symptom onset within 70 days of vaccination could have been missed. However, this would have led to a bias toward finding an increased risk in the (primary) SCRI analysis, in which a Days 43–70 control interval was used for Doses 3 and 4. Yet no statistically significant elevated risk was found. In effect, the shorter follow-up period strengthens the null result. Moreover, the post hoc sensitivity analysis using a Days 29–56 control interval for all doses also produced null results. The study was not powered to assess the risk of Kawasaki disease by dose number.

In summary, we found no evidence of an elevated risk of Kawasaki disease in the 4 weeks after PCV13 vaccination nor any evidence of an elevated risk extending or concentrated beyond 4 weeks. The consistency of the results across alternative designs, age-adjustment methods, control intervals, and levels of case confirmation included suggests that the null findings are highly robust.

## Supporting information

S1 STROBE Checklist STROBE checklist of items that should be included in reports of observational studies. (DOCX)

S1 Protocol Study protocol. (DOCX)

S1 Data Analysis dataset for SCRI analysis using confirmed Kawasaki disease cases and Doses 1 and 2 control window = Days 29–56 and Doses 3 and 4 control window = Days 43–70. SCRI, self-controlled risk interval.
(XLS)

S2 Data Analysis data set for SCRI analysis using confirmed and possible Kawasaki disease cases and Doses 1 and 2 control window = Days 29–56 and Doses 3 and 4 control window = Days 43–70. SCRI, self-controlled risk interval.
(XLS)

S3 Data Analysis data set for SCRI analysis using confirmed Kawasaki disease cases and all control windows = Days 29–56 and no cases for which (KD admit–KD onset) > 14 days. KD, Kawasaki disease; SCRI, self-controlled risk interval.
(XLS)

S4 Data Analysis data set for SCRI analysis using confirmed and possible Kawasaki disease cases and all control windows = Days 29–56 and no cases for which (KD admit–KD onset) > 14 days. KD, Kawasaki disease; SCRI, self-controlled risk interval.
(XLS)

## Abbreviations

CDC: Centers for Disease Control and Prevention
CPT: Current Procedural Terminology
FDA: Food and Drug Administration
HCPCS: Healthcare Common Procedure Coding System
HCUP: Healthcare Cost and Utilization Project
ICD-9: International Classification of Diseases 9th revision
ICD-10: International Classification of Diseases 10th revision
NDC: National Drug Code
PCV: pneumococcal conjugate vaccine
PCV13: 13-valent pneumococcal conjugate vaccine
PRISM: Postlicensure Rapid Immunization Safety Monitoring
RR: relative risk
SCRI: self-controlled risk interval
