# Supplementary material for: Kawasaki disease and 13-valent pneumococcal conjugate vaccination among young children: A self-controlled risk interval and cohort study with null results
Source: PLoS Med. 2019 Jul 2;16(7):e1002844. doi: 10.1371/journal.pmed.1002844 (PMC6605647; doi:10.1371/journal.pmed.1002844)
Supplement: S1 Protocol — (DOCX) [file pmed.1002844.s002.docx]

**CBER** **Sentinel PRISM Surveillance Protocol**

KAWASAKI DISEASE AND PCV13 VACCINE

**Prepared by:** Meghan A. Baker, MD, ScD,^1^ Bethany Baer, MD,^2^ Martin Kulldorff, PhD,^3^ Megan Reidy, MPH,^1^ Carolyn Balsbaugh, MPH,^1^ Grace M. Lee, MD, MPH,^1^ Sandra Feibelmann, MPH,^1^ David Cole, BM,^1^ W. Katherine Yih, PhD, MPH^1^

**Author Affiliations:** 1. Department of Population Medicine, Harvard Pilgrim Health Care Institute and Harvard Medical School, Boston, MA; 2. Center for Biologics Evaluation and Research, Food and Drug Administration, Silver Spring, MD; 3. Division of Pharmacoepidemiology and Pharmacoeconomics, Brigham and Women’s Hospital, Boston, MA

**Version 1 September 15, 2015**

**Version 2 August 16, 2016**

Sentinel is sponsored by the [U.S. Food and Drug Administration (FDA)](http://www.fda.gov/) to monitor the safety of FDA-regulated medical products. Sentinel is one piece of the [Sentinel Initiative](http://www.fda.gov/Safety/FDAsSentinelInitiative/default.htm), a multi-faceted effort by the FDA to develop a national electronic system that complements previously existing methods of safety surveillance. Sentinel Collaborators include Data and Academic Partners that provide access to healthcare data and ongoing scientific, technical, methodological, and organizational expertise. The Sentinel Coordinating Center is funded by the FDA through the Department of Health and Human Services (HHS) Contract number HHSF223201400030I

| **Version** | **Date** | **Modification** | **By** |
| --- | --- | --- | --- |
| V2 | 08/16/2016 | - Clarified that this evaluation does not assume that there is an association between PCV13 exposure and Kawasaki disease. - Deleted objective to explore the role of concomitant vaccines. - Included the ICD-10 code based algorithm in addition to ICD-9 code based algorithm for identifying Kawasaki disease. Clarified in the text that we will evaluate the positive predictive values of the ICD-9 and ICD-10 code based algorithms for Kawasaki disease separately and combined together. - Increased the number of Data Partners from 4 to 6. - For the study population, both exposed and unexposed to PCV vaccine, we further explained the number of days continuously enrolled in the Data Partner. - Removed CPT code for PCV7 vaccination and added CPT code for unspecified PCV vaccine exposure code. - For the outcome definition, added the ICD-10 code for acute febrile mucocutaneous lymph node syndrome and explained codes in the inpatient setting will be in any position (primary diagnosis, secondary diagnosis, etc.). - For the outcome definition, further explained what the first code in 365 days in the inpatient setting will include. - Changed the number of days following all doses of PCV13 vaccines from 38 to 42 days in order to identify cases whose symptoms began within the 28 day risk window. Also specified the risk window applies to PCV13 as opposed to PCV. - For the self-controlled analysis, added that diagnoses that occur up to 14 days beyond the end of the control window will be captured. Also explained how cases will be classified. There will be no censoring of person-time within risk windows and control windows. - Clarified that the unadjusted analysis in the self-controlled analysis will use logistic regression. - For the Cohort Poisson Regression Analysis, exposed person-time will include from the time of licensure of PCV13 (February 2010) to the most up-to-date data. - Attempting to include state/zip code as a proxy for race/ethnicity in the model for the Cohort Poisson Regression Analysis has been removed. - For the Cohort Poisson Regression Analysis, the unexposed time period has been clarified. - A secondary cohort analysis has been added with a defined risk interval to further explore findings from a prior study regarding vaccination associated with Kawasaki disease incidence. - State/zip code as a surrogate for race/ethnicity has been removed from the section on potential confounders and effect modifiers. - Deleted Exploring the Role of Concomitant Vaccines section. - ICD-10 code M30.3 for acute febrile mucocutaneous lymph node syndrome has been added to the automated code analysis section. - Deleted the Post-study Combined Analysis section - A reference for a prior study (Abrams et al.) has been added. | Sentinel Operations Center Kawasaki Disease and PCV13 Vaccine Workgroup |

**CBER Sentinel PRISM Surveillance Protocol**

Kawasaki Disease and PCV13 Vaccine

**Table of Contents**

I. Introduction - 1 -

II. Background - 1 -

A. Public Health Significance and Study Motivation - 1 -

B. PCV13 - 2 -

C. Kawasaki Disease - 3 -

III. Objectives - 5 -

IV. Methods - 5 -

A. Study Population and Data Sources - 5 -

B. Exposure Codes - 6 -

C. Outcome Definition - 6 -

D. Risk Window - 6 -

E. Statistical AnalysEs - 7 -

1. Descriptive analysis - 7 -

2. Overview of hypothesis-testing analyses - 7 -

3. Self-controlled analysis - 7 -

4. Cohort Poisson Regression Analysis - 9 -

5. Potential confounders and effect modifiers - 10 -

6. Statistical power - 10 -

7. Evaluation of Elevated Risks - 12 -

8. Automated code analysis - 12 -

F. Medical Chart Review - 12 -

V. Dataset Creation - 14 -

VI. References - 15 -

# Introduction

The Post-Licensure Rapid Immunization Safety Monitoring (PRISM) program was established in August 2009 as one of several 2009 H1N1 influenza vaccine safety surveillance efforts launched and supported by the federal government. In September 2010, PRISM was incorporated into what was then Sentinel’s pilot project, Mini-Sentinel, to provide FDA with an active surveillance capability for vaccines to inform regulatory decision-making. The transition to Sentinel occurred in FY2015. To further explore a potential safety signal seen in the Vaccine Adverse Event Reporting System (VAERS),[^1^](#_ENREF_1) this protocol describes an effort to evaluate whether an increased risk of Kawasaki disease is associated with receipt of the 13-valent pneumococcal conjugate vaccine (PCV13). If an association is observed, then its magnitude will be quantified.

# Background

## Public Health Significance and Study Motivation

Pre-licensure trials of both PCV7 (Prevnar; Wyeth) and PCV13 (Prevnar13; Wyeth) vaccines found no increased risk for serious adverse events.[^2^](#_ENREF_2) In a post-licensure safety study unadjusted analysis, however, an increase in Kawasaki disease hospitalizations among PCV7 vaccinated patients was reported when compared to historical controls.[^3^](#_ENREF_3) Further analysis adjusted for sex, race, age at first dose, length of follow-up and season, found no evidence for an association of Kawasaki disease with the PCV7 vaccination.[^3^](#_ENREF_3) Hua et al. explored 29 reports of Kawasaki disease reported for PCV7 vaccination to the Vaccine Adverse Events Reporting System (VAERS) from 1990-2007. The evaluation did not suggest an elevation of the risk of Kawasaki disease following PCV7.[^4^](#_ENREF_4) Post-licensure surveillance of the PCV13 vaccine in the Vaccine Safety Datalink (VSD) compared the cumulative risk of Kawasaki disease within 28 days of vaccination with PCV13 to the historical risk within 28 days of vaccination with PCV7 from 2007 to 2009. A statistical signal for Kawasaki disease after PCV13 was identified at the second group sequential test (of the 12 group sequential tests over a 90-week period that had been planned for each of the 7 health outcomes of interest). Subsequently, the investigators conducted a single end-of-study analysis of Kawasaki disease, in which they obtained medical records to adjudicate each case identified by automated Kawasaki disease diagnoses. Using confirmed cases only, the risk within 0-28 days following vaccination with PCV13 when compared to PCV7 was 2.38 (0.92, 6.38). This result was not found to be statistically significant and was not controlled for race/ethnicity. The study authors noted that the possible association between PCV13 and Kawasaki disease, as suggested by the elevated relative risk estimate, may deserve further investigation.[^5^](#_ENREF_5) A review of the first 18 months of licensure of PCV13 was completed by the FDA under the FDA Amendment Act of 2007 section 915. This review included an analysis of the PCV13 VSD study results as well as an evaluation of the VAERS proportional reporting ratios (PRR) for Kawasaki disease. The review resulted in an FDA internet posting that reported there had been adverse event reports of Kawasaki disease following administration of PCV13. This 915 posting also stated that the FDA intended to initiate a larger study of Kawasaki disease risk following PCV13 vaccination in PRISM.[^1^](#_ENREF_1)

The present protocol describes the study that will be conducted to fulfill the FDA commitment to further explore the possible relationship between PCV13 vaccination and Kawasaki disease. This evaluation does not a priori assume that there is an association between PCV13 exposure and Kawasaki disease.

## PCV13

Infection by *Streptococcus pneumoniae* is identified by the World Health Organization (WHO) as a major cause of pneumonia, bacteremia and meningitis. Although over 90 pneumococcal serotypes have been identified, a small subset of serotypes account for the majority of disease.[^6^](#_ENREF_6) Prior to licensure of the pneumococcal conjugate vaccine, young children were highly susceptible to pneumococcal disease, with an estimated 17,000 cases of invasive disease and 200 resulting deaths occurring annually in children ≤5 years of age in the United States. An additional 5 million cases per year of acute otitis media were believed to result from pneumococcal disease in children ≤5 years of age.[^7^](#_ENREF_7)

In 2000, FDA licensed the first pneumococcal conjugate vaccine, 7-valent pneumococcal vaccine (PCV7) (Prevnar; Wyeth), for use in infants and young children. PCV7 protected against strains of *Streptococcus pneumoniae* that most commonly caused illness among children in the U.S. Subsequently, inclusion of PCV7 in the recommended children immunization program resulted in decreased rates of invasive pneumococcal disease.[^8^](#_ENREF_8) The CDC reported that rates of PCV7-type invasive pneumococcal disease in children under 5 years of age dropped from 80 cases per 100,000 to less than 1 case per 100,000 following the implementation of the PCV7 vaccine.[^9^](#_ENREF_9) FDA licensed a second vaccine, PCV13 on February 24^th^, 2010 to protect against 6 additional serotypes that accounted for much of the invasive pneumococcal disease in young children not covered by the PCV7 vaccine.[^10^](#_ENREF_10) The Advisory Committee on Immunization Practices (ACIP) recommended PCV13 for use as a 4-dose vaccination series, administered at 2, 4, 6 and 12-15 months of age, similar to the PCV7 vaccine recommendations. Full implementation of the transition from PCV7 to PCV13 came into effect in March 2010 with supplies of PCV13 available to the public sector that month as shown in Figure 1.[^11^](#_ENREF_11) By July 2010, Pfizer reported that >90% of its private shipments of pneumococcal conjugate vaccines were for PCV13.[^11^](#_ENREF_11) Children previously vaccinated with PCV7 finished their remaining vaccinations with PCV13. A dose of PCV13 was recommended by ACIP for children 14-59 months of age who had completed the 4-dose vaccination series with PCV7, and children with specified underlying medical conditions receive a 5^th^ dose until 71 months of age.[^10^](#_ENREF_10)


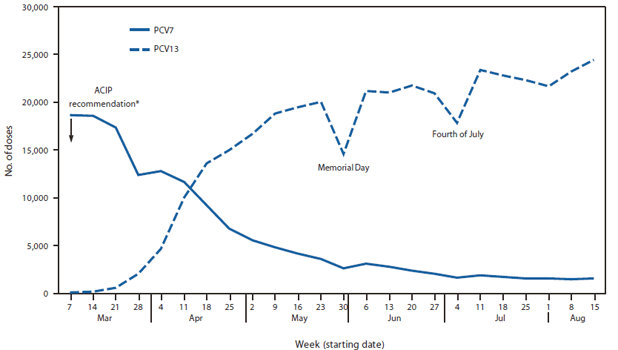


Figure 1. “Pneumococcal conjugate vaccine doses administered to children aged 0 through 59 months, by vaccine type and week --- Immunization Information System sentinel sites, United States, March 7--August 21, 2010.” [^11^](#_ENREF_11)

## Kawasaki Disease

Kawasaki disease is an acute, self-limited vasculitis and is the leading cause of acquired heart disease in children in the United States. Kawasaki disease primarily affects infants and young children, as shown in Figure 2 and Figure 3. The etiology of Kawasaki disease is unknown, although theories include an infectious cause or immunologic response triggered by an infectious agent.[^12^](#_ENREF_12)^,^[^13^](#_ENREF_13) No disease-specific laboratory test is available. Hence, the diagnosis of Kawasaki disease is based on a clinical case definition as described by the American Heart Association, which specifies that patients have fever lasting for ≥5 days (or fever until the date of administration of intravenous immunoglobulin) and at least 4 of the following 5 primary clinical features.[^13^](#_ENREF_13)

1. Changes in the extremities (erythema of palms or soles; edema of hands or feet; periungual peeling of fingers or toes)
2. Polymorphous exanthem rash
3. Bilateral conjunctival injection without exudates
4. Changes in lips and oral cavity (inflamed lips or throat, strawberry tongue, or dry/cracking lips)
5. Cervical lymphadenopathy (at least 1 lymph node ≥ 1.5 cm in diameter)

“Incomplete” or “atypical” Kawasaki disease can also be diagnosed in patients presenting with fever for ≥5 days and <4 of the clinical features, if coronary artery disease is diagnosed using echocardiography or coronary angiography.[^13^](#_ENREF_13) The diagnosis of incomplete Kawasaki disease is more common in infants <6 months of age, and these patients are more likely to develop coronary artery abnormalities as compared with infants between 6 and 12 months of age.[^13-15^](#_ENREF_13) In general, physicians are encouraged to consider a diagnosis of Kawasaki disease in febrile patients with fewer clinical features of Kawasaki disease in the absence of cardiac complications for prompt treatment to reduce the risk of coronary artery aneurysms.[^16^](#_ENREF_16) If left untreated, approximately 25% of patients develop coronary artery aneurysms which may lead to additional cardiac complications including myocardial infarction, sudden death or ischemic heart disease.[^13^](#_ENREF_13)^,^[^17^](#_ENREF_17)^,^[^18^](#_ENREF_18) Intravenous immunoglobulin administration has been identified as a successful treatment in reducing cardiac complications in Kawasaki disease patients if promptly administered within 10 days of illness onset. Aspirin is also used in the treatment of Kawasaki disease for the anti-inflammatory and anti-platelet effects.

Numerous factors have been shown to affect the incidence of Kawasaki disease in the United States. The illness presents most commonly in Americans of Asian and Pacific Island descent, with an incidence of 32.5/100,000 in children <5 years of age compared to 9.1/100,000 for non-Hispanic whites of the same age group.[^13^](#_ENREF_13)^,^[^19^](#_ENREF_19) In addition, Kawasaki disease is age-dependent, with 80% of cases occurring before age 5 years, and a peak incidence at 13-24 months of age.[^20^](#_ENREF_20) Many studies have reported increased incidence of Kawasaki disease in males, with male to female ratios ranging from 1.5-1.7 : 1.[^4^](#_ENREF_4)^,^[^7^](#_ENREF_7)^,^[^19^](#_ENREF_19)^,^[^21^](#_ENREF_21)^,^[^22^](#_ENREF_22) Seasonality is also believed to have some impact on incidence.[^19^](#_ENREF_19)


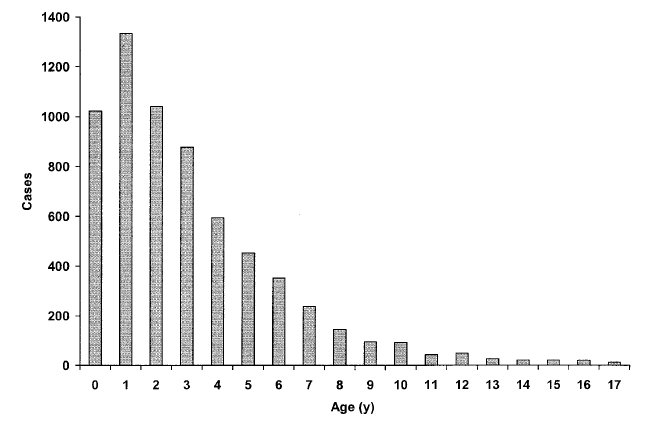


Figure 2. “Number of cases in the National Inpatient Sample (NIS) data presented by patients’ age. The number of cases peaks at patients 1 to 2 years of age, followed by patients <1 year and 2 to 3 years of age, and then the number continues to decrease towards older age.”[^23^](#_ENREF_23)


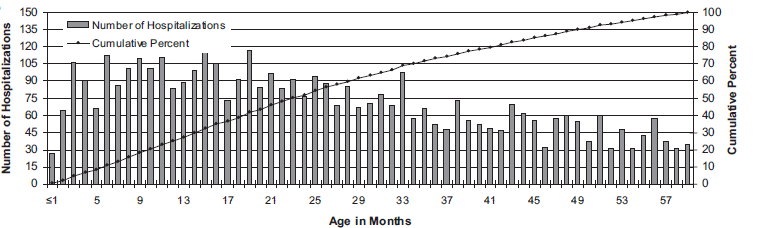
Figure 3. “Kawasaki syndrome associated hospitalizations and cumulative percent by … age in months for children <5 years, United States, 2006.”[^24^](#_ENREF_24)

# Objectives

The objectives of this study are:

1. To determine the existence and magnitude of any increased risk of Kawasaki disease in the 28 days following PCV13 vaccination.
2. To determine through medical chart review the positive predictive value of an ICD-9 and ICD-10 code based algorithm for identifying Kawasaki disease.

# Methods

## Study Population and Data Sources

Six Data Partners will participate in this evaluation, and all will have data available from 2008 until the most recent data refresh. The study population will include infants and children from birth until 23.99 months of age who were members of the participating Data Partners during 2008 to 2015 and who meet the other enrollment criteria. When the data analysis programs are run, we will use the most up-to-date information from each Data Partner.

Infants and children meeting the following criteria will be included in the evaluation:

- Exposed to at least 1 dose of PCV vaccine and continuously enrolled in the Data Partner from birth through at least 84 days (risk and control intervals (56 days) plus 28 days to account for delays in symptom onset and Kawasaki disease diagnosis coding) after their first dose of PCV vaccine.
- Unexposed to PCV vaccine and continuously enrolled in the Data Partner from birth through at least 144 days of age (60 days, the time that many infants would receive dose 1, plus 84 days), with at least one documented healthcare visit in any setting between 14 and 150 days.

Gaps of up to 45 days between birth and enrollment will be allowed. For both exposed and unexposed categories of subjects, continuously enrolled person-time through a maximum age of 23.99 months will be included in the analyses, with the following proviso:

- If an infant in the exposed category has incomplete person-time during *0-84 days after Dose 2* of PCV vaccine, his/her person-time on and after the day of that vaccination will be excluded;
- If a child in the exposed category has incomplete person-time during *0-98 days after Dose 3 or 4* of PCV vaccine, his/her person-time on and after the day of that vaccination will be excluded.

## Exposure Codes

- PCV13 vaccination will be identified by the CPT code 90670 and the NDC codes 00005197101, 00005197102, 00005197104, 00005197105.
- PCV 7 vaccination will be identified by the NDC codes 00005197049, 00005197050, 00005197067, 54569511700.
- Unspecified PCV vaccine will be identified by HCPCS codes G0009, S0195 and the CPT code 90669. (The latter code is technically for PCV7, but we will treat it as “unspecified” PCV in order to guard against possible misclassification of PCV13 as PCV7.)

Unspecified PCV vaccine prior to February 24, 2010 will be assumed to be PCV7 and after August 2010 will be assumed to be PCV13 based on the date of approval for PCV13 and the fact that by July 2010, Pfizer reported that >90% of its private shipments of pneumococcal conjugate vaccines were for PCV13.[^11^](#_ENREF_11) Information related to unspecified PCV vaccines received between Feb 24 and Aug 31, 2010, will not be included in analyses. Data on all vaccinations occurring within the first two years of life will also be captured.

## Outcome Definition

Cases of Kawasaki disease will be identified by the ICD-9 code 446.1 and ICD-10 code M30.3 (acute febrile mucocutaneous lymph node syndrome) in the inpatient setting in any position (primary diagnosis, secondary diagnosis, etc.). We assume that a confirmed or suspected case of Kawasaki disease would be admitted to a hospital to initiate treatment or undergo further clinical evaluation. Only the first code in 365 days in the inpatient setting for patients at least 365 days of age or first ever for those under 365 days of age will be considered in order to exclude follow-up visits for Kawasaki disease.

## Risk Window

Risk windows will include 1-28 days following all doses of PCV13 vaccines. This is the same risk window that was used in the VSD evaluation of PCV13 vaccine and is supported by evidence that among siblings, more than one half of second Kawasaki disease cases in each family developed within 10 days of onset of symptoms in the first case.[^5^](#_ENREF_5)^,^[^25^](#_ENREF_25) We will evaluate diagnoses within 42 days following all doses of PCV13 vaccines in order to identify cases whose symptoms began within the 28 day risk window even if their diagnosis was not made or recorded until later. Cases will be classified as occurring in the risk or control window based on the day that symptoms began. The control window is discussed in the next section.

## Statistical AnalysEs

### Descriptive analysis

Descriptive analyses using automated claims data will be performed to characterize the PCV7 and PCV13 vaccination data and Kawasaki disease data. They will include frequency distributions of age at the time of receipt of the vaccines stratified by Data Partner, sex and year, and frequency distributions of the number of Kawasaki disease cases by age.

### Overview of hypothesis-testing analyses

A challenge in studying PCV13 and Kawasaki disease will be the confounding effect of age, as both vaccination and the risk of Kawasaki disease are age-dependent. To confront this challenge, we will use both a *self-controlled risk interval (SCRI)*[*^26^*](#_ENREF_26)*^,^*[*^27^*](#_ENREF_27) and a *cohort* design. A major advantage of the former, which we are pre-specifying as the primary method, is that it inherently controls for all fixed (non-time-varying) potential confounders such as gender, race/ethnicity, and chronic predisposing conditions, by virtue of each subject serving as his/her own control. Another advantage of the SCRI design is that it uses only exposed cases, thus minimizing potential misclassification bias due to incomplete data on vaccine exposure. A possible disadvantage is that adjustment for time-varying confounders such as age must be made explicitly, and estimation of the age effect may introduce error. The cohort design is likely to have higher statistical power than the self-controlled design, due to the relatively large amount of historical and concurrent unexposed data involved in the generation of expected counts. However, its ability to control for time-invariant confounding is not as good as that of the self-controlled approach. If there are differences among racial/ethnic groups in vaccine coverage and timing, an important potential confounder in the cohort design will be race/ethnicity. The point estimates are also subject to bias from misclassification of exposure if some vaccinations are not recorded in the claims data and not captured during the chart review process.

### Self-controlled analysis

We will use a self-controlled risk interval (SCRI) design with a risk interval of Days 1-28 post-vaccination and a 28-day-long comparison (control) interval following this risk interval (Figure 4). We will capture diagnoses that occur up to 14 days beyond the end of the control window in order to identify cases in which symptoms began within the control window. Cases will be classified as occurring in the risk or control window based on the day that symptoms began. The null hypothesis is that the risk of Kawasaki disease is the same during the 28 days after PCV13 vaccine as it is during the subsequent 28–day-long control period. Because the recommended interval between doses 1 and 2 and between doses 2 and 3 is 2 months, there is less time in which to position the control interval than for the subsequent doses. The control interval will be Days 29-56 following doses 1 and 2. For doses 3 and 4, it will be Days 43-70 post-vaccination (Figure 4). (The latter is preferable due to uncertainty about the true period of vaccine-associated risk, which, if it exists, might extend beyond 28 days after vaccination.) There will be no censoring of person-time within risk and control windows. Only individuals with an observation in either the risk or the control interval are informative for this self-controlled analysis. In the final report, we will provide the following descriptive information, summed over all Data Partners, but separate for each dose: (i) total number of cases in the risk interval, (ii) total number of cases in the control interval, and (iii) a histogram of the total number of cases by age in weeks at the time of vaccination.

Kawasaki disease

Dose 1 Kawasaki disease Dose 2 Dose 3 and 4

**1 28 29 56 1 28 29 56 1 28 43 70**

risk control risk control risk control

Figure 4. Self-controlled risk interval design to evaluate the incidence rate ratios comparing rates in risk vs. control intervals.

We will run both unadjusted and age adjusted analyses. The unadjusted analysis will use logistic regression. We will employ two methods for adjustment of age.

Our primary analysis will use risk-by-age estimates from the literature to estimate the risk of Kawasaki disease by week of age. Specifically, we will use the smoothed estimates from Holman et al.’s data on hospitalizations for Kawasaki Syndrome in the United States.[^24^](#_ENREF_24) With such an external estimate, age enters the regression through an offset term in the regression equation. With one observation per individual, we will use logistic regression, where an observation is one if the outcome is in the risk interval and zero if it is in the control interval. Unlike in the unadjusted analysis, we will include an offset term to adjust for the differential risk of Kawasaki disease at the ages in the risk and comparison intervals. This offset term requires a good estimate of the natural relative incidence of Kawasaki disease by age. It will be treated in this logistic regression analysis as known with certainty, which has been shown to work well in other studies (Li et al 2015, manuscript in progress).

In a secondary analysis, we will adjust for age with conditional Poisson regression. The outcome will be the occurrence of Kawasaki disease. We will compare the risk of Kawasaki disease during the 28-day risk interval after PCV13 to the risk of Kawasaki disease during the 28-day comparison interval, adjusting for the four-week age difference between these two intervals. The risk of Kawasaki disease by week of age will be modeled as a continuous function using either polynomials or splines. The selection of the best-fitting polynomial or splines to describe the risk-by-age relationship will be done without including the risk interval in the model, to ensure integrity of the results. This method will estimate the incidence curve by age from PRISM Kawasaki disease rates in all eligible exposed or unexposed time. In the Poisson regression, the conditioning will be done on individuals, ensuring a self-controlled analysis. This means that individuals without the outcome in either the risk or the control interval are only used to estimate the incidence curve by age, while individuals with the outcome in either the risk or control interval are used to estimate both the incidence curve and the risk of Kawasaki disease associated with the vaccine.

Because the sample size to be used for the age adjustment is larger in the Holman et al. study than it is expected to be in our study population, the first method is preferable, in our view. Note that, if the incidence estimate is off by the same magnitude at all ages, for example, 10% higher than actual at all ages, it is inconsequential for a self-controlled analysis.

Study design and analysis methods are shown in Table 1.

### Cohort Poisson Regression Analysis

The second design that we will use is a cohort design with unconditional Poisson regression, including the exposed person-time from the time of licensure of PCV13 (February 2010) to the most up-to-date data, and unexposed person-time for the entire cohort from the year 2008 to the most up-to-date data. The following covariates will be included in versions of the Poisson regression model before settling on the most parsimonious and explanatory model: vaccination risk window (yes/no), age in weeks modeled as a continuous variable, sex, and calendar year as a continuous variable, and Data Partner. It is not feasible to include race/ethnicity as a covariate in this analysis as that information is not widely available in the Sentinel data. Exposed person-time will be the 1-28 days after PCV13 vaccination, while unexposed time will be the time outside of the 7 days before through the 42 days after vaccination with either PCV13 or PCV7. (We exclude the 7 days prior to vaccination from unexposed time in order to control for the healthy vaccinee effect and similar effects that could result from dependency of vaccination on one’s health condition in the week prior.) We will include unexposed person-time from children who are not vaccinated with PCV7 or PCV13 but have at least one documented healthcare visit. A polynomial risk function will be used to refine estimates of background rates by week of age during the first 1.5 years of life. We will systematically try at least linear, quadratic, cubic, fourth-order, and fifth-order functions, without exposure in the model (to blind ourselves to the effect of vaccination), and select the function to include in the final model based on the log-likelihood ratio, p-value, Akaike information criterion (AIC), and biologic plausibility. If we cannot find a well-fitting polynomial function, we will try splines instead.

Dose PCV13 Kawasaki disease

unexposed exposed unexposed

Figure 5. Cohort design to evaluate the incidence rate ratios comparing rates in the exposed and unexposed intervals

In addition to the results from the Poisson regression, the final report will include the total number of observed and expected cases during the exposed time, summed over all the data providers. Stratified by dose and age in weeks, but summed over all data providers, we will also present a table with the number of cases during exposed time, the number of cases during unexposed time, the number of exposed person-days and the number of unexposed person-days. In a secondary cohort analysis, we will consider the exposed interval as 1-42 days after PCV13 vaccination and unexposed time as the time outside of the 7 days before vaccination with either PCV13 or PCV7 through the 42 days after vaccination. We include this risk interval to further explore the finding in a study by Abrams et al. suggesting that vaccination was associated with a transient decrease in Kawasaki disease incidence.^28^

Table 1. Study designs and analyses. The primary analysis is the self-controlled logistic regression with age adjustment using an incidence curve from the literature.

| **Study designs and analyses** | | | |
| --- | --- | --- | --- |
| Self-controlled | | | Cohort |
| Logistic regression | | Conditional Poisson regression | Unconditional Poisson regression |
| **Primary analysis:**  Age adjustment using offset term based on incidence curve from literature | No age adjustment | Age adjustment internal, based on polynomial risk function from study population | Age adjustment internal, using polynomial or spline risk function |

### Potential confounders and effect modifiers

We will collect information on both time-variant and time-invariant factors considering the multiple study designs. Race/ethnicity is controlled for in the self-control analysis but will be a possible confounder in the cohort analysis. While season does appear to be associated with Kawasaki disease,[^19^](#_ENREF_19) we do not expect it to be associated with vaccination status or timing. Viral and bacterial illnesses have been suggested as possible etiological agents of Kawasaki disease. ^24,29^ When available in the medical chart, we will capture information on infections occurring in the 60 days prior to onset of Kawasaki disease. Additionally, PCV13 is generally given concomitantly with multiple other vaccines and this can be a source of confounding.

### Statistical power

One-sided power calculations were done for the self-controlled risk interval analysis, alpha=0.05, and all PCV13 doses. PCV13 dose counts were obtained from four Data Partners in March, 2015. Background rates of ICD-9 code identified Kawasaki disease were estimated by counts in three Data Partners as we expect that background rates would be consistent among the Data Partners. The results of the self-controlled risk interval power calculations, assuming all potential cases are chart confirmed, are shown in Table 2 below. There will be 80% power to detect a relative risk of 1.9.

Table 2. Power calculation based on a total 3,388,104 PCV13 doses

| **Power calculation for the self-controlled risk interval design** | | |
| --- | --- | --- |
| Kawasaki disease diagnosis/100,000 Person Years | Relative Risk | Power |
| 8.7 | 1.1 | 9.3 |
| 8.7 | 1.2 | 15.5 |
| 8.7 | 1.3 | 23.6 |
| 8.7 | 1.4 | 33.1 |
| 8.7 | 1.5 | 43.5 |
| 8.7 | 1.6 | 53.8 |
| 8.7 | 1.8 | 72.3 |
| 8.7 | 1.9 | 80.0 |
| 8.7 | 2.0 | 85.5 |
| 8.7 | 2.1 | 90.0 |
| 8.7 | 2.5 | 98.3 |
| 8.7 | 3.0 | 99.9 |
| 8.7 | 4.0 | 100.0 |
| 8.7 | 5.0 | 100.0 |

If we assume 80% chart retrieval and 50% chart confirmation (0.4 overall, or 3.5 confirmed cases/100,000 person-years), based in part on past experience with chart-review studies in PRISM, the relative risk that could be detected with 80% power would be between 2.5 and 3.0, as shown in Table 3.

Table 3. Power calculation based on the above but assuming only 40% of the potential cases are ultimately confirmed.

| **Power calculation for the self-controlled risk interval design (0.4 of confirmed cases in Table 2)** | | |
| --- | --- | --- |
| Kawasaki disease diagnosis/100,000 Person Years | Relative Risk | Power |
| 3.5 | 1.1 | 7.5 |
| 3.5 | 1.2 | 10.7 |
| 3.5 | 1.3 | 14.5 |
| 3.5 | 1.4 | 19.0 |
| 3.5 | 1.5 | 24.0 |
| 3.5 | 1.6 | 29.4 |
| 3.5 | 1.8 | 41.0 |
| 3.5 | 1.9 | 47.3 |
| 3.5 | 2.0 | 52.7 |
| 3.5 | 2.1 | 58.3 |
| 3.5 | 2.5 | 76.8 |
| 3.5 | 3.0 | 90.7 |
| 3.5 | 4.0 | 99.0 |
| 3.5 | 5.0 | 99.9 |

### Evaluation of Elevated Risks

If a statistically significant elevated risk is found in any of the analyses, we will perform the following:

1. Check data quality, descriptive statistics and background rates by age, sex and Data Partner.
2. Use the temporal scan statistic to test for temporal clustering of chart-confirmed Kawasaki disease cases in the weeks after the dose in question.

### Automated code analysis

As an exploratory objective, if the positive predictive value of the ICD-9 code 446.1 and ICD-10 code M30.3 (acute febrile mucocutaneous lymph node syndrome) in the inpatient setting is ≥0.8 for Kawasaki disease among the chart confirmed cases, we will report the automated code analyses in addition to the chart reviewed analyses. For the automated code analyses, we will estimate day of symptom onset based on the median number of days from onset to diagnosis for the cases that are chart reviewed.

## Medical Chart Review

Medical records of all cases of inpatient diagnosis of Kawasaki disease, limited to the first inpatient diagnosis in 365 days, occurring during eligible person-time in the study period will be reviewed, regardless of vaccination status or timing relative to vaccination. The maximum total number of cases to be reviewed is 200 among all Data Partners combined. If the available number of cases exceeds 200, we will use a pre-specified sampling scheme to ensure that the highest-priority cases are reviewed. Top priority cases are those that fall within either the risk or control window of the self-control designs. All of these will be chart reviewed. High priority cases include the remaining cases falling between 0 and 18 months of age and during the period of time when PCV13 was administered, starting from the most recent time period and moving back in time. A chart abstraction form will be used.

In order to identify the cases and obtain the medical charts, we will send programs for the Data Partners to run on their uniform-format patient-level files. These programs will produce a report of the number and characteristics (e.g., age and sex) of the cases and, for each case, a report listing the healthcare encounters occurring within a specified number of days of the Kawasaki disease diagnosis, to be specified after the first review of the descriptive characteristics. The reports will include information on clinical setting, actual diagnosis, and date of the diagnosis.

Next, PRISM clinical investigators will rank the encounters of each case using relevant diagnosis and procedure codes provided in the report to identify those that seem likely to produce the most definitive diagnostic information. The ranked list will be returned to the Data Partners who will then attach patient name, insurance member number, and provider name and address to all visits. Another PRISM program to be run at the Data Partners will organize the list of charts to pull by facility.

Each Data Partner will identify a preferred vendor to create chart extracts. These chart extracts will consist of components pre-specified by the PRISM team that need to be photocopied or scanned by the vendor. Examples of such items include the admission note, the daily notes during hospitalization, the discharge summary, all cardiology reports within 12 weeks of the index date (defined as the first day of fever or the date of the first code for Kawasaki disease if the first date of fever is missing) and all diagnostic procedures, such as echocardiograms and angiograms. Using the list provided by the Data Partner, the chart-review vendor will notify the facilities, contact them to obtain the charts, photocopy or scan the appropriate pages of the chart, and redact the record of all personal identifiers. The Data Partners will have the option of reviewing the redacted records to ensure that the redaction is complete. The redacted records will be sent to the Sentinel Operations Center for further review and abstraction by the PRISM team.

All investigators classifying the cases will be blinded to vaccination status. PRISM clinical investigators will review the chart abstractions and classify the cases. Two clinical investigators will independently review 10 charts, blinded to vaccination history as well as to the other reviewer’s decision. This initial round of case classification will enable refinement of the classification rules. Using the refined set of rules, investigators will complete a second round of case classification for another 10 cases. If there are zero discrepancies between reviewers after the second round, then the review will continue to use a single reviewer for the remainder of the cases. If there are any discrepancies regarding the 10 second-round cases, double review of each subsequent case will be required with regular consensus review involving both reviewers. If consensus cannot be reached, a third party reviewer will make the final determination.

Diagnosis of Kawasaki disease will be based on the American Heart Association Diagnosis and the Centers for Disease Control and Prevention guidelines.[^13^](#_ENREF_13)^,30^ Our primary diagnosis for Kawasaki disease will include patients meeting the following criteria:

- ≥4 principal features and a fever (≥38.0˚C) persisting ≥5 days *or* fever until the date of administration of IVIG if given before the 5^th^ day of fever
- Evidence of < 4 principal features and coronary artery disease (aneurysm or dilation) by echocardiography or coronary angiography *and* fever (≥38.0˚C) of any duration.

Possible Kawasaki disease will be defined as evidence of 2 or 3 principal features and ≥ 5 days of fever. A large proportion of our study population will be under the age of 1 year, and some principal clinical features may frequently be absent in young infants.[^13^](#_ENREF_13) A sensitivity analysis will be conducted including possible Kawasaki disease in the case definition.

For cases of Kawasaki disease without a prior PCV vaccination history, the immunization record will be sought from the child’s primary care provider to verify that the patient was not vaccinated with a PCV vaccine or correct the absence of the vaccination record.

The positive predictive value of the Kawasaki disease ICD-9 and ICD-10 code to identify confirmed cases will be determined for all the cases captured by the definition, stratified by Data Partner, PCV13 vaccine dose, and timing relative to PCV13 vaccination. We will evaluate the positive predictive values of the ICD-9 and ICD-10 code based algorithms for Kawasaki disease separately and combined together.

# Dataset Creation

We will use the Sentinel distributed Common Data Model (MSCDM), which allows Data Partners to maintain control over patient-level data. Data Partners extract and output data into eight files of standard format. The files relevant for the present study are: enrollment, demographic, encounter, procedure, diagnosis and dispensing.

Sentinel programmers will provide Data Partners with programs to be run on the patient-level files. The programs will produce aggregate data on vaccinations and Kawasaki disease organized in strata defined by variables such as week of vaccination, type of vaccine, dose number, age, Data Partner, and sex, with counts of patients, vaccine doses and Kawasaki disease in particular strata. Data Partners will return the aggregate data for analysis at Harvard, using Sentinel’s secure file transport methods.

# References

1. U.S. Food and Drug Administration, Postmarket Drug Safety Evaluation Summaries Completed from January 2012 through June 2012. (Accessed 5/8/2015, at <http://www.fda.gov/Drugs/GuidanceComplianceRegulatoryInformation/Surveillance/ucm355034.htm>)

2. Nuorti JP, Whitney CG. Prevention of pneumococcal disease among infants and children - use of 13-valent pneumococcal conjugate vaccine and 23-valent pneumococcal polysaccharide vaccine - recommendations of the Advisory Committee on Immunization Practices (ACIP). MMWR Recommendations and reports : Morbidity and mortality weekly report Recommendations and reports / Centers for Disease Control 2010;59:1-18.

3. Center KJ, Hansen JR, Lewis E, Fireman BH, Hilton B. Lack of association of Kawasaki disease after immunization in a cohort of infants followed for multiple autoimmune diagnoses in a large, phase-4 observational database safety study of 7-valent pneumococcal conjugate vaccine: lack of association between Kawasaki disease and seven-valent pneumococcal conjugate vaccine. The Pediatric infectious disease journal 2009;28:438-40.

4. Hua W, Izurieta HS, Slade B, et al. Kawasaki disease after vaccination: reports to the vaccine adverse event reporting system 1990-2007. The Pediatric infectious disease journal 2009;28:943-7.

5. Tseng HF, Sy LS, Liu IL, et al. Postlicensure surveillance for pre-specified adverse events following the 13-valent pneumococcal conjugate vaccine in children. Vaccine 2013.

6. World Health Organization (WHO), Pneumococcal disease. 2014. (Accessed 8/4/2015, 2015, at <http://www.who.int/immunization/diseases/pneumococcal/en/.>)

7. Han RK, Sinclair B, Newman A, et al. Recognition and management of Kawasaki disease. CMAJ : Canadian Medical Association journal = journal de l'Association medicale canadienne 2000;162:807-12.

8. Invasive pneumococcal disease in children 5 years after conjugate vaccine introduction--eight states, 1998-2005. MMWR Morbidity and mortality weekly report 2008;57:144-8.

9. Centers for Disease Control and Prevention, Pneumococcal. 2014. (Accessed 8/3/2015, 2015, at [http://www.cdc.gov/vaccines/pubs/surv-manual/chpt11-pneumo.html](http://www.cdc.gov/vaccines/pubs/surv-manual/chpt11-pneumo.html.))

10. Licensure of a 13-valent pneumococcal conjugate vaccine (PCV13) and recommendations for use among children - Advisory Committee on Immunization Practices (ACIP), 2010. MMWR Morbidity and mortality weekly report 2010;59:258-61.

11. Invasive pneumococcal disease and 13-valent pneumococcal conjugate vaccine (PCV13) coverage among children aged </=59 months---selected U.S. regions, 2010--2011. MMWR Morbidity and mortality weekly report 2011;60:1477-81.

12. Nakamura Y, Yashiro M, Uehara R, Oki I, Watanabe M, Yanagawa H. Monthly observation of the number of patients with Kawasaki disease and its incidence rates in Japan: chronological and geographical observation from nationwide surveys. Journal of epidemiology / Japan Epidemiological Association 2008;18:273-9.

13. Newburger JW, Takahashi M, Gerber MA, et al. Diagnosis, treatment, and long-term management of Kawasaki disease: a statement for health professionals from the Committee on Rheumatic Fever, Endocarditis and Kawasaki Disease, Council on Cardiovascular Disease in the Young, American Heart Association. Circulation 2004;110:2747-71.

14. Burns JC, Wiggins JW, Jr., Toews WH, et al. Clinical spectrum of Kawasaki disease in infants younger than 6 months of age. The Journal of pediatrics 1986;109:759-63.

15. Rosenfeld EA, Corydon KE, Shulman ST. Kawasaki disease in infants less than one year of age. The Journal of pediatrics 1995;126:524-9.

16. Red Book, Kawasaki Disease. In: Kimberlin DW, Brady MT, Jackson MA, Long SS, eds. Red Book: American Academy of Pediatrics; June, 2015:494-500.

17. Kato H, Sugimura T, Akagi T, et al. Long-term consequences of Kawasaki disease. A 10- to 21-year follow-up study of 594 patients. Circulation 1996;94:1379-85.

18. Dajani AS, Taubert KA, Gerber MA, et al. Diagnosis and therapy of Kawasaki disease in children. Circulation 1993;87:1776-80.

19. Holman RC, Belay ED, Curns AT, Schonberger LB, Steiner C. Kawasaki syndrome hospitalizations among children in the United States, 1988-1997. Pediatrics 2003;111:448.

20. Gedalia A. Kawasaki disease: an update. Current rheumatology reports 2002;4:25-9.

21. Luca NJ, Yeung RS. Epidemiology and management of Kawasaki disease. Drugs 2012;72:1029-38.

22. Mason WH, Takahashi M. Kawasaki syndrome. Clinical infectious diseases : an official publication of the Infectious Diseases Society of America 1999;28:169-85; quiz 86-7.

23. Chang RK. Hospitalizations for Kawasaki disease among children in the United States, 1988-1997. Pediatrics 2002;109:e87.

24. Holman RC, Belay ED, Christensen KY, Folkema AM, Steiner CA, Schonberger LB. Hospitalizations for Kawasaki syndrome among children in the United States, 1997-2007. The Pediatric infectious disease journal 2010;29:483-8.

25. Fujita Y, Nakamura Y, Sakata K, et al. Kawasaki disease in families. Pediatrics 1989;84:666-9.

26. Kramarz P, DeStefano F, Gargiullo PM, et al. Does influenza vaccination exacerbate asthma? Analysis of a large cohort of children with asthma. Vaccine Safety Datalink Team. Archives of family medicine 2000;9:617-23.

27. Klein NP, Hansen J, Lewis E, et al. Post-marketing safety evaluation of a tetanus toxoid, reduced diphtheria toxoid and 3-component acellular pertussis vaccine administered to a cohort of adolescents in a United States health maintenance organization. The Pediatric infectious disease journal 2010;29:613-7.

28. Abrams JY, Weintraub ES, Baggs JM et al. Childhood vaccines and Kawasaki disease, Vaccine Safety Datalink, 1996-2006. Vaccine 2015;33:382-7.

29. Kao AS, Getis A, Brodine S, Burns JC. Spatial and temporal clustering of Kawasaki syndrome cases. The Pediatric infectious disease journal 2008;27:981-5.

30. Centers for Disease Control and Prevention, Kawasaki Syndrome. (Accessed 05/13/15, at <http://www.cdc.gov/kawasaki/case-definition.html>)
